# Construction and Comprehensive Analysis of a circRNA-miRNA-mRNA Regulatory Network to Reveal the Pathogenesis of Hepatocellular Carcinoma

**DOI:** 10.3389/fmolb.2022.801478

**Published:** 2022-01-24

**Authors:** Meile Mo, Bihu Liu, Yihuan Luo, Jennifer Hui Juan Tan, Xi Zeng, Xiaoyun Zeng, Dongping Huang, Changhua Li, Shun Liu, Xiaoqiang Qiu

**Affiliations:** ^1^ Department of Epidemiology, School of Public Health, Guangxi Medical University, Nanning, China; ^2^ Department of Acute Care Surgery, The First Affiliated Hospital of Guangxi Medical University, Nanning, China; ^3^ Yong Loo Lin School of Medicine, National University of Singapore, Singapore, Singapore; ^4^ Department of Occupational and Environmental Health, School of Public Health, Guilin Medical University, Guilin, China; ^5^ Department of Sanitary Chemistry, School of Public Health, Guangxi Medical University, Nanning, China; ^6^ Department of Maternal, Child and Adolescent Health, School of Public Health, Guangxi Medical University, Nanning, China

**Keywords:** hepatocellular carcinoma, circular RNA, microarray, bioinformatics analysis, circRNA-miRNA-mRNA network

## Abstract

**Background:** Circular RNAs (circRNAs) have been demonstrated to be closely related to the carcinogenesis of human cancer in recent years. However, the molecular mechanism of circRNAs in the pathogenesis of hepatocellular carcinoma (HCC) has not been fully elucidated. We aimed to identify critical circRNAs and explore their potential regulatory network in HCC.

**Methods:** The robust rank aggregation (RRA) algorithm and weighted gene co-expression network analysis (WGCNA) were conducted to unearth the differentially expressed circRNAs (DEcircRNAs) in HCC. The expression levels of DEcircRNAs were validated by quantitative real-time polymerase chain reaction (qRT-PCR). A circRNA-miRNA-mRNA regulatory network was constructed by computational biology, and protein-protein interaction (PPI) network, functional enrichment analysis, survival analysis, and infiltrating immune cells analysis were performed to uncover the potential regulatory mechanisms of the network.

**Results:** A total of 22 DEcircRNAs were screened out from four microarray datasets (GSE94508, GSE97332, GSE155949, and GSE164803) utilizing the RRA algorithm. Meanwhile, an HCC-related module containing 404 circRNAs was identified by WGCNA analysis. After intersection, only four circRNAs were recognized in both algorithms. Following qRT-PCR validation, three circRNAs (hsa_circRNA_091581, hsa_circRNA_066568, and hsa_circRNA_105031) were chosen for further analysis. As a result, a circRNA-miRNA-mRNA network containing three circRNAs, 17 miRNAs, and 222 mRNAs was established. Seven core genes (*ESR1*, *BUB1*, *PRC1*, *LOX*, *CCT5*, *YWHAZ*, and *DDX39B*) were determined from the PPI network of 222 mRNAs, and a circRNA-miRNA-hubgene network was also constructed. Functional enrichment analysis suggested that these seven hub genes were closely correlated with several cancer related pathways. Survival analysis revealed that the expression levels of the seven core genes were significantly associated with the prognosis of HCC patients. In addition, we also found that these seven hub genes were remarkably related to the infiltrating levels of immune cells.

**Conclusion:** Our research identified three pivotal HCC-related circRNAs and provided novel insights into the underlying mechanisms of the circRNA-miRNA-mRNA regulatory network in HCC.

## Introduction

Primary liver cancer is a common malignant tumour and the third leading cause of cancer-related deaths worldwide, threatening our health and life. Hepatocellular carcinoma (HCC) is the most common type of liver cancer, accounting for 75–80% of cases (Sung et al., 2021). Although great improvements have been achieved in the diagnosis and treatment of HCC, most patients diagnosed with advanced HCC have limited treatment options and their 5-years survival rate is less than 15% (Bertuccio et al., 2017). Besides, HCC patients with surgical resection are still suffering from high risk of recurrence and lung metastasis (Kulik et al., 2018). Given the great perniciousness of HCC, there is an urgent need to explore the molecular cascades of the development and progression of HCC.

Circular RNAs (circRNAs), a class of endogenous non-coding RNAs, were first discovered in viroids in 1976 (Sanger et al., 1976). Unlike traditional linear RNAs, circRNAs are characterized by their covalently closed-loop structures with neither the 5′ end cap nor 3′ poly (A) tails, enabling them to be resistant to the digestion of RNase R (Suzuki et al., 2006). In the past, circRNAs were generally considered to be products of aberrant RNA splicing due to their low abundance and lack of poly (A) tails. However, with the recent developments in high-throughput sequencing technology, circRNAs have been found to be closely related to many human diseases including cancer, cardiovascular diseases, autoimmune diseases, Alzheimer’s disease, and so on (Meng et al., 2017; Altesha et al., 2019; Zhou et al., 2019; Huang J.-L. et al., 2020).

A large body of evidence have showed that circRNAs function through a variety of ways, such as by regulating transcription, splicing and chromatin interactions, acting as microRNA (miRNA) decoys, serving as scaffolds for circRNA–protein complexes, being translated to proteins, and so on (Chen, 2020; Shi et al., 2020; Zhou et al., 2020). Emerging evidence revealed that abundant conserved miRNA response elements (MREs) exist in circRNAs so that the circRNAs can function as competing endogenous RNAs (ceRNAs) in the regulation of human cancer progression (Cui et al., 2018; Zhong et al., 2018). For example, circRNF20 harbours miR-487a to regulate the expression of HIF-1α and HK2, thereby promoting breast cancer tumorigenesis (Cao et al., 2020). CircNRIP1 can promote gastric cancer and cervical cancer progression by sponging miR-149-5p (Zhang et al., 2019) and miR-629-3p (Li X. et al., 2020), respectively. Research have also shown that circHIPK3 regulates cancer cell growth by sponging multiple miRNAs, such as miR-7 (Zeng et al., 2018), miR-485-3p (Lai et al., 2020), miR-107 (Wei J. et al., 2020), and so on. Furthermore, numerous circRNAs have been demonstrated to play crucial roles in HCC progression through acting as ceRNAs (Han et al., 2020; Xu G. et al., 2020). However, the circRNA-related ceRNA network in HCC have not been completely elucidated and further studies are needed.

**FIGURE 1:**
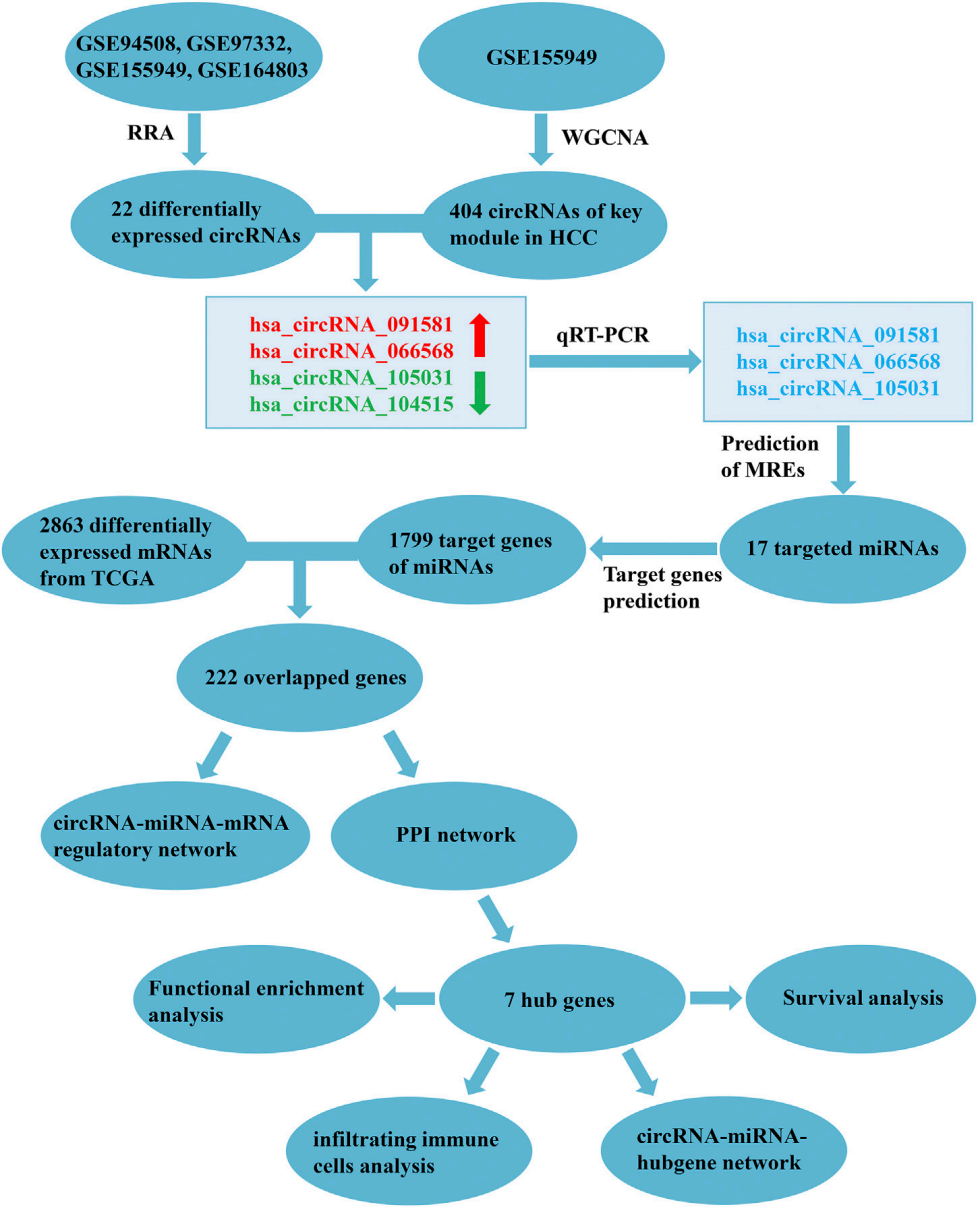
Flowchart of the present study. RRA, robust rank aggregation; WGCNA, weighted gene co-expression network analysis; HCC, hepatocellular carcinoma; qRT-PCR, quantitative real-time polymerase chain reaction; MREs, miRNA response elements; TCGA, the Cancer Genome Atlas; PPI, protein-protein interaction.

In the present study, we mined the pivotal circRNAs and constructed the circRNA-related ceRNA regulatory network in HCC using the Gene Expression Omnibus (GEO) database, the Cancer Genome Atlas (TCGA) database, and through bioinformatics methods. The flow chart of our study is exhibited in Figure 1. Firstly, robust rank aggregation (RRA) (Kolde et al., 2012) and weighted gene co-expression network analysis (WGCNA) (Langfelder and Horvath, 2008) algorithms were applied to find out the HCC-related circRNAs based on GEO datasets. Then, the expression levels of selected circRNAs were verified by quantitative real-time polymerase chain reaction (qRT-PCR). Subsequently, computational biology was utilized to predict the potential circRNA-miRNA-mRNA interactions. A protein-protein interaction (PPI) network was constructed to identify the hub genes of the ceRNA network. Finally, functional enrichment analysis, survival analysis, and immune cell infiltration analysis of hub genes were performed to thoroughly figure out the potential mechanisms of the ceRNA network.

## Materials and Methods

### Human Tissue Specimens

In our study, 30 pairs of HCC and their corresponding paracancerous tissues were collected from the Affiliated Cancer Hospital of Guangxi Medical University. All patients were diagnosed with HCC based on pathological examination and did not receive any preoperative anticancer treatment. The tissues were snap frozen in liquid nitrogen after isolation then stored in liquid nitrogen until usage. All patients signed informed consent forms and our study was permitted by the Ethics Committee of Guangxi Medical University.

### Expression Data Collected From the GEO and TCGA Database

Microarray datasets of circRNA expression profile in HCC tissues were retrieved from the GEO database (http://www.ncbi.nlm.nih.gov/geo/) using the following keywords: (circular RNA OR circRNA) AND (hepatocellular carcinoma OR HCC OR liver cancer). The datasets selected met the following filter criteria of 1) The circRNA expression profile was derived from cancer tissues and normal tissues in HCC patients; 2) The sample size was at least five for each group. Eventually, four microarray datasets (GSE94508, GSE97332, GSE155949, and GSE164803) were enrolled and further analyzed.

The RNA sequencing (RNA-seq) data of HCC tissues and normal tissues was extracted from the Cancer Genome Atlas Liver Hepatocellular Carcinoma (TCGA-LIHC) project (https://cancergenome.nih.gov/). The expression data were obtained and converted into transcripts per million (TPM), and then log2 transformation was used for normalization. Finally, mRNA expression profile of 371 HCC tissues and 50 adjacent normal tissues were utilized for differential expression analysis.

### Differential Expression and RRA Analysis

First, the microarray dataset (GSE94508) with raw counts were normalized by the “edgeR” package (version: 3.34.0). Then, the “limma” package (version: 3.48.1) was implemented to screen the differentially expressed circRNAs (DEcircRNAs) and mRNAs (DEmRNAs) of the four GEO datasets and TCGA database, respectively. DEcircRNAs and DEmRNAs were defined as those with a *p*-value < 0.05 and the |log2 (fold change)| > 1. Volcano plots of the differential expression results were generated using the “ggplot2” package (version: 3.3.5). In order to integrate and rank all the DEcircRNAs of these four circRNA microarray datasets, the “RobustRankAggreg” package (version: 1.1) was conducted for RRA analysis (Kolde et al., 2012). The heatmap for the result of RRA analysis was created by the “pheatmap” package (version: 1.0.12) in R.

### WGCNA Analysis

Considering that the GSE155949 dataset has the largest sample size (49 pairs of HCC tissues and matched adjacent normal tissues) and the largest number of detected circRNAs (n = 10592) simultaneously, GSE155949 was selected for WGCNA analysis. The “WGCNA” package (version: 1.70–3) was used to investigate the relationships between gene modules and clinical traits (Langfelder and Horvath, 2008). In the present study, the optimum soft threshold power was six when we set the scale free *R* ^2^ > 0.8. After the adjacency matrix was converted into a Topological Overlap Matrix (TOM), the circRNAs were clustered into different module eigengenes (MEs) when the minimal module size was set to 30. Then, the correlations between MEs and HCC were calculated using the Pearson correlation analysis and the module with the highest correlation coefficient was selected for further analysis.

### qRT-PCR, TA Cloning and Sequencing

Total RNA was extracted from tissues using the Trizol Reagent (Invitrogent, USA) and reverse transcribed to complementary DNA using the PrimeScript™ RT reagent Kit with gDNA Eraser (Takara, Japan) following the manufacturer’s instructions. The relative RNA expression level was evaluated by the TB GreenTM Premix Ex TaqTM II Kit (Takara, Japan) in the real-time PCR system (Applied Biosystems StepOnePlus, USA). *GAPDH* was employed as the internal control. The primer sequences are listed in Table 1. To verify whether the PCR products of the four circRNAs contain their back-splice sites, the amplified products were cloned into a TA vector (Zero TOPO-TA Cloning Kit, Sangon Biotech) and sequenced by Sangon Biotech (Shanghai, China) Co., Ltd. The relative expression levels of the four circRNAs were calculated using the 2^−△△Ct^ method.

**TABLE 1:** Primer sequences of RNAs for qRT-PCR.

Gene names	Primer sequence (5′-3′)	
	Forward	Reverse
GAPDH	AGC​CAC​ATC​GCT​CAG​ACA​C	GCC​CAA​TAC​GAC​CAA​ATC​C
hsa_circRNA_091581	GAC​CAC​CAC​TAG​GCC​TTT​GAA	TGG​AGT​CAG​GCT​TGG​GTA​GT
hsa_circRNA_066568	TGA​CTG​TTC​AAG​ATA​CGA​GGA​GG	CTC​TCA​CGT​TCC​CCA​ACC​AT
hsa_circRNA_105031	GAT​GCA​CGG​TGC​TAC​ACC​TA	GGA​AAA​GAA​CCC​TGA​TTG​CCT​G
hsa_circRNA_104515	TGA​ACC​TGA​CAC​CAT​CAG​CAA	TGA​GTC​TGA​GGC​CAC​TCC​TT
GPC3	CAG​TCA​GCA​GGC​AAC​TCC​GAA​G	AAG​AAG​AAG​CAC​ACC​ACC​GAG​ATG
ROBO1	TGT​TGC​TTT​GGG​ACG​GAC​TGT​AAC	GGC​TGG​ATG​ACT​GTG​GTG​GTT​G
MBNL3	CTG​ATT​CCT​GGA​AAC​CCA​CCT​CTT​G	CAC​GCT​GAA​ATT​CTC​GGC​AAA​CC
AGK	AGT​GTC​TCC​AAG​CCA​GCC​AGT​G	CTC​CAC​AGG​CAT​CGC​TTC​ATA​CTC

qRT-PCR, quantitative real-time polymerase chain reaction.

### RNase R Treatment

Total RNA (5 µg) was incubated at 37°C for 15 min with or without 4U/µg of RNase R (Epicentre Biotechnologies, USA), followed by RNase R inactivation at 70°C for 15min. After that, reverse transcription and qRT-PCR were conducted to detect the expression levels of the four circRNAs and their corresponding mRNAs. *GAPDH* expression in the RNase R (-) group was used as reference, and the expression levels of target RNAs were presented as mean ± standard deviation.

### Construction of the circRNA-miRNA-mRNA Regulatory Network

Firstly, the target binding miRNAs of circRNAs were predicted by two online websites: circBank (http://www.circbank.cn/index.html) (Liu et al., 2019) and Circular RNA Interactome (CircInteractome, https://circinteractome.nia.nih.gov/) (Dudekula et al., 2016). Only miRNAs predicted by both websites were regarded as potential target miRNAs. As for the prediction of miRNA-mRNA pairs, TargetScanHuman 7.2 (http://www.targetscan.org/vert_72/) (Agarwal et al., 2015), miRWalk 3.0 (http://mirwalk.umm.uni-heidelberg.de/) (Sticht et al., 2018), miRTarBase (https://mirtarbase.cuhk.edu.cn/∼miRTarBase/miRTarBase_2022/php/index.php) (Huang H.-Y. et al., 2020), and miRDB (http://mirdb.org/) (Chen and Wang, 2020) were applied. Target mRNAs predicted by at least three databases were selected. Subsequently, the overlapping genes of the predicted target mRNAs and DEmRNAs were considered as candidate interest genes to construct the circRNA-miRNA-mRNA regulatory network. The ceRNA network was visualized by the Cytoscape software (version: 3.8.2).

### Establishment of the PPI Network and Identification of Hub Genes

To elucidate the interaction among the candidate interest genes, a PPI network was constructed by the STRING (https://www.string-db.org/) (Szklarczyk et al., 2019) online tool and visualized by the Cytoscape software. The size of the network nodes indicated the concatenation degree: the larger the nodes, the higher the connection degree. The edges’ size demonstrated the combined score of two interacting genes, with a thicker line representing a greater combined score. To identify the hub genes of the PPI network, the degree of mRNAs was calculated by the Cytoscape plugin “cytoHubba”, and mRNAs with a degree ≥10 were regarded as hub genes.

### Functional Enrichment Analysis

DIANA-miRPath v3.0 (http://snf-515788.vm.okeanos.grnet.gr/) (Vlachos et al., 2015) was employed to investigate the Kyoto Encyclopedia of Genes and Genomes (KEGG) pathways in which the predicted miRNAs may be involved, and *p* < 0.05 was considered statistically significant. Gene Ontology (GO) annotation, KEGG pathway, and Reactome pathway enrichment analyses of hub genes were carried out using the KOBAS-i (http://kobas.cbi.pku.edu.cn/) (Bu et al., 2021) online tool. Pathways with corrected *p* value <0.01 were selected in our study.

### Survival Analysis of Hub Genes

To further explore the influence of hub genes on the prognosis, GEPIA2 (http://gepia2.cancer-pku.cn/#index) (Li et al., 2021) was implemented to determine the relationships between hub genes and the overall survival (OS) and disease-free survival (DFS) of HCC patients. In the present study, Kaplan-Meier plots were drawn and hub genes with log-rank *p* < 0.05 were considered statistically significant. The hazard ratio (HR) of high expression group compared with low expression group could also be obtained from the survival analysis.

### Association Between Core Genes and Infiltrating Immune Cells in HCC

TIMER2.0 (http://timer.cistrome.org/) (Li T. et al., 2020) is a comprehensive resource for systematic analysis of immune infiltrates across diverse cancer types. We used the TIMER web tool to investigate the correlations between the hub genes and infiltrating immune cells, including B cells, CD8^+^ T cells, CD4^+^ T cells, neutrophils, macrophages, and myeloid dendritic cells. *p*-value less than 0.05 was considered statistically significant.

## Results

### Identification of 22 DEcircRNAs in HCC Based on the RRA Method

**FIGURE 2:**
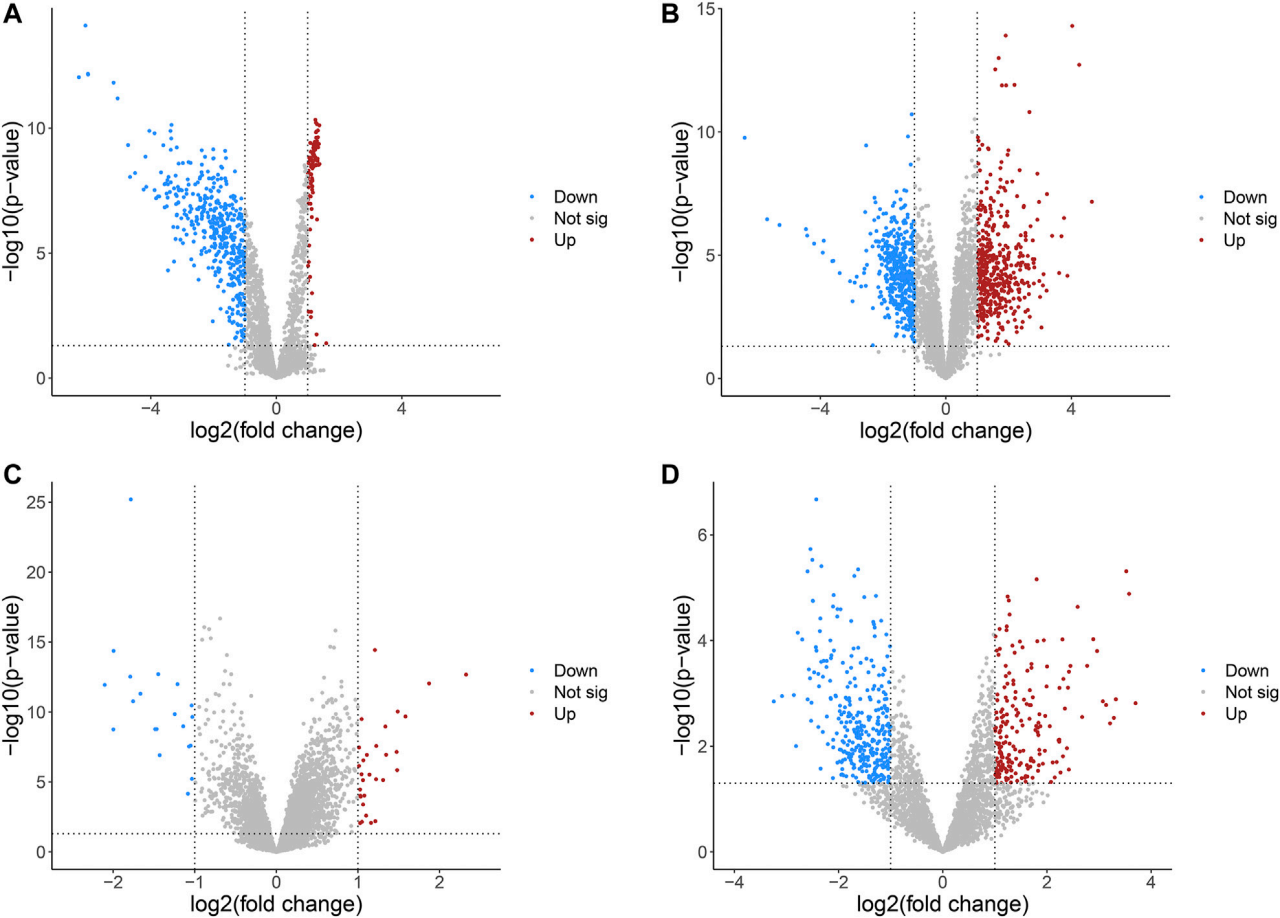
Volcano plots of differentially expressed circRNAs in HCC from four GEO datasets. **(A)** GSE94508. **(B)** GSE97332. **(C)** GSE155949. **(D)** GSE164803. HCC, hepatocellular carcinoma; GEO, Gene Expression Omnibus.

**FIGURE 3:**
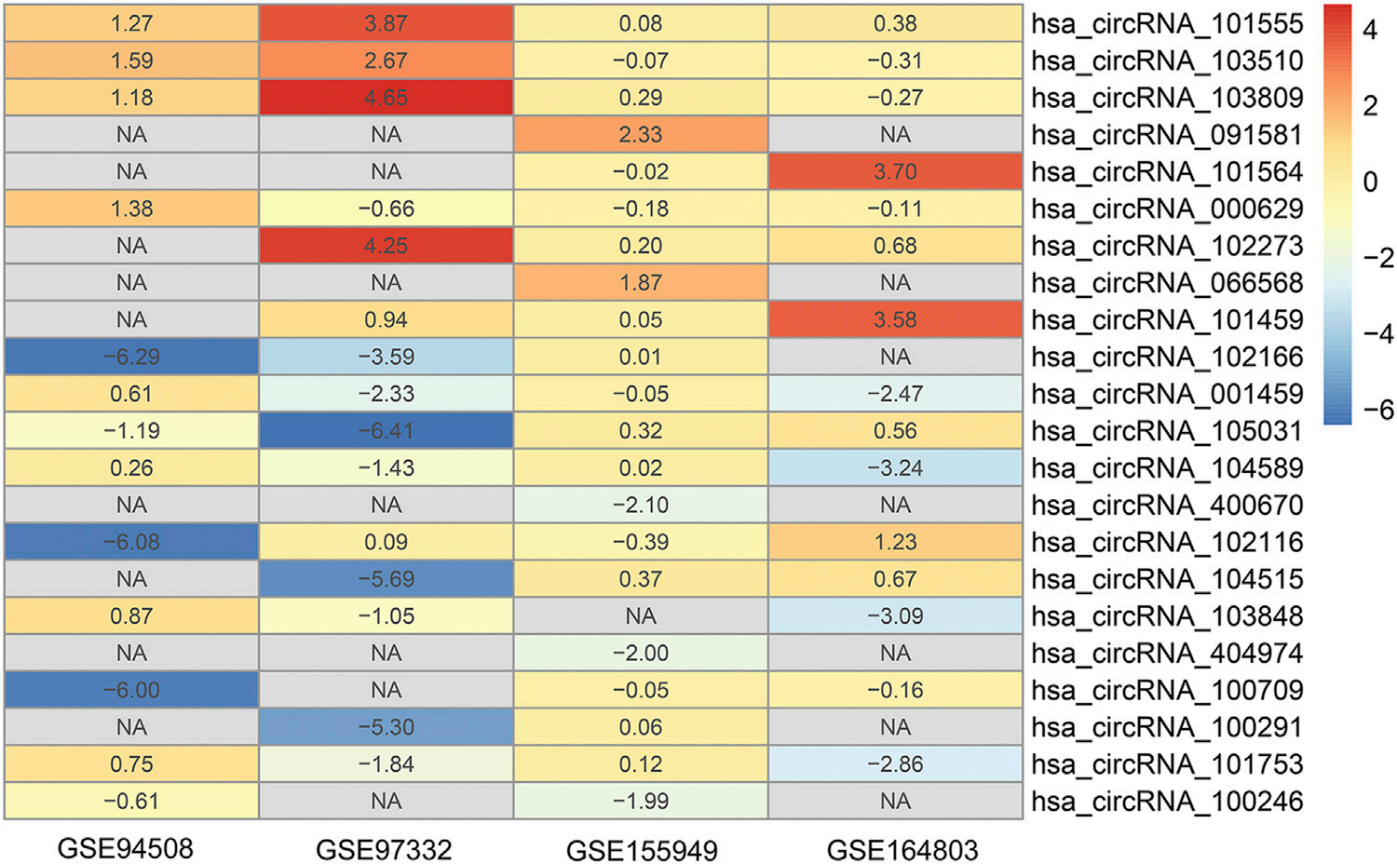
Heatmap of the 22 differentially expressed circRNAs identified by the robust rank aggregation algorithm.

The detailed information of four included circRNA microarray datasets (GSE94508, GSE97332, GSE155949, and GSE164803) is listed in Table 2. In brief, 523 DEcircRNAs were identified in the GSE94508 dataset, including 96 upregulated circRNAs and 427 downregulated circRNAs (Figure 2A). A total of 892 DEcircRNAs with 453 upregulated circRNAs and 439 downregulated circRNAs were found in the GSE97332 dataset (Figure 2B). Meanwhile, 50 DEcircRNAs consisting of 30 upregulated circRNAs and 20 downregulated circRNAs were determined in the GSE155949 dataset (Figure 2C). As for the GSE164803 dataset, 514 DEcircRNAs, of which 193 were upregulated and 321 were downregulated, were recognized (Figure 2D). After that, we integrated and ranked the DEcircRNAs from the four microarray chips using the RRA algorithm. Finally, 22 DEcircRNAs, including nine upregulated circRNAs and thirteen downregulated circRNAs, were identified to be statistically significant with a *p*-value < 0.05 (Figure 3).

**TABLE 2:** The detailed information of the four circRNA microarray datasets from GEO database.

GEO datasets	Year	Platform	First author	Country	Sample size (T/N)	Number of circRNAs	Number of DEcircRNAs	Upregulated circRNAs	Downregulated circRNAs
GSE94508	2017	GPL19978	Fu L	China	5/5	2572	523	96	427
GSE97332	2017	GPL19978	Han D	China	7/7	3,471	892	453	439
GSE155949	2021	GPL21825	Han J	Singapore	49/49	10592	50	30	20
GSE164803	2021	GPL19978	Bing Y	China	6/6	3,054	514	193	321

GEO, gene expression omnibus; T, tumour; N, normal.

### Identification of Key circRNA Co-Expression Module Based on WGCNA Analysis

**FIGURE 4:**
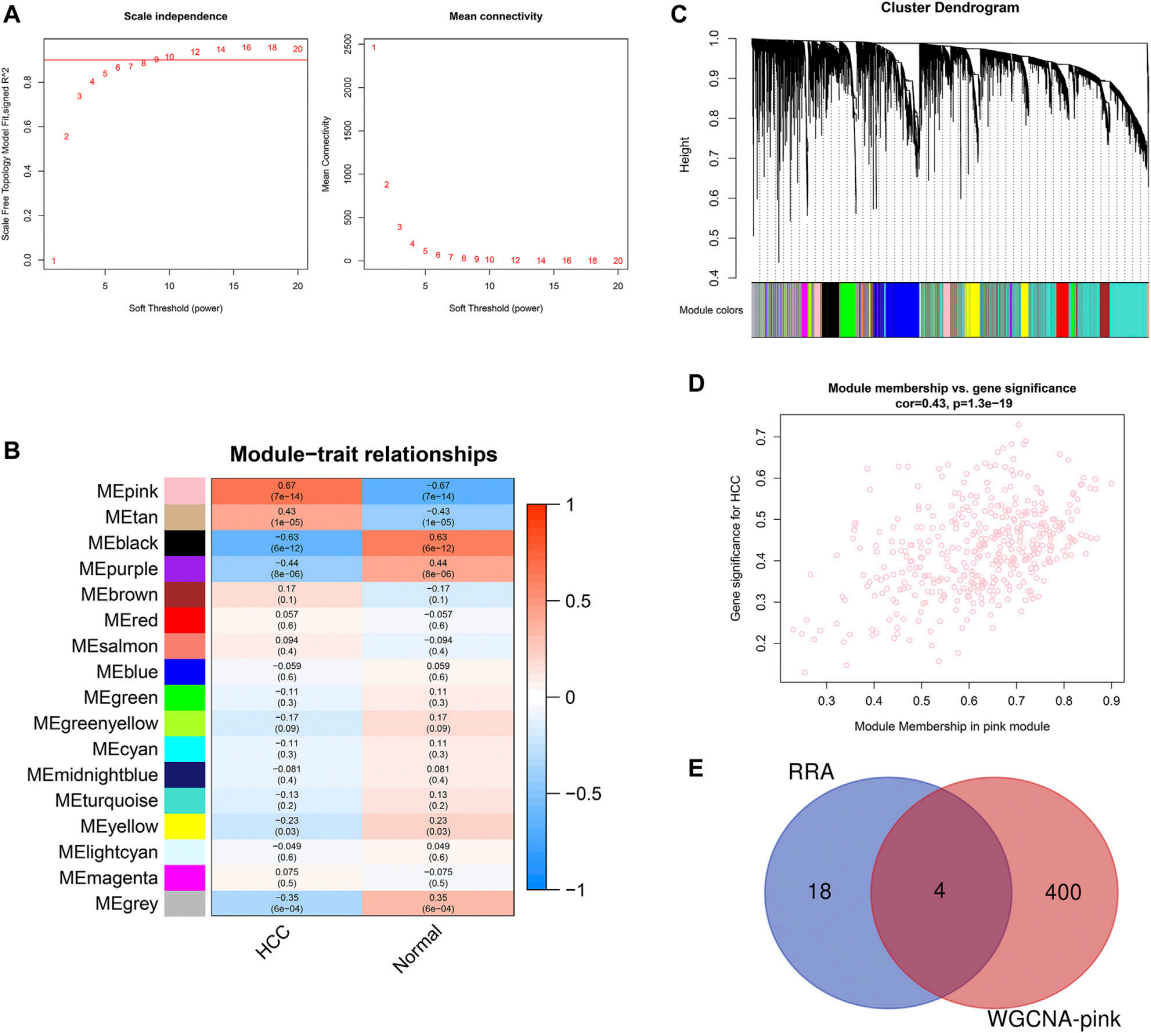
Identification of key module associated with HCC in the GSE155949 dataset using WGCNA analysis. **(A)** Determination of the best soft threshold (power). When power = 6, the scale-free *R* ^2^ > 0.8 and mean connectivity <100. **(B)** Cluster dendrogram of co-expression circRNAs modules. **(C)** Relationships between different modules and HCC tissues. The corresponding correlation coefficient and *p*-value are presented in each cell. **(D)** Scatter plot of gene significance and module membership in the pink module. **(E)** Venn diagram of the overlapping circRNAs identified by the RRA algorithm and WGCNA analysis. RRA, robust rank aggregation; WGCNA, weighted gene co-expression network analysis.

To identify the key module of circRNA in HCC, WGCNA analysis was conducted in the GSE155949 dataset. When setting the scale-free *R* ^2^ > 0.8 and mean connectivity of all circRNAs <100, six was chosen as the best soft threshold (power) in our study (Figure 4A). A total of 17 co-expression modules were classified using the cluster analysis and the cluster dendrogram is presented in Figure 4B. Subsequently, the relationships between the modules and HCC were calculated and is shown in Figure 4C. The pink module containing 404 circRNAs had the strongest positive correlation (*r* = 0.67, *P* = 7e-14) with the HCC tissues. Besides, the scatter plot of the module membership vs. gene significance in the pink module also showed a significant correlation (*r* = 0.43, *p* = 1.3e-19; Figure 4D). Then, these 404 circRNAs of the pink module overlapped with 22 DEcircRNAs identified by the RRA method, and only four circRNAs (hsa_circRNA_091581, hsa_circRNA_066568, hsa_circRNA_105031, and hsa_circRNA_104515) were recognized in both algorithms (Figure 4E).

### Validation of the Expression of Four circRNAs Using qRT-PCR

**FIGURE 5:**
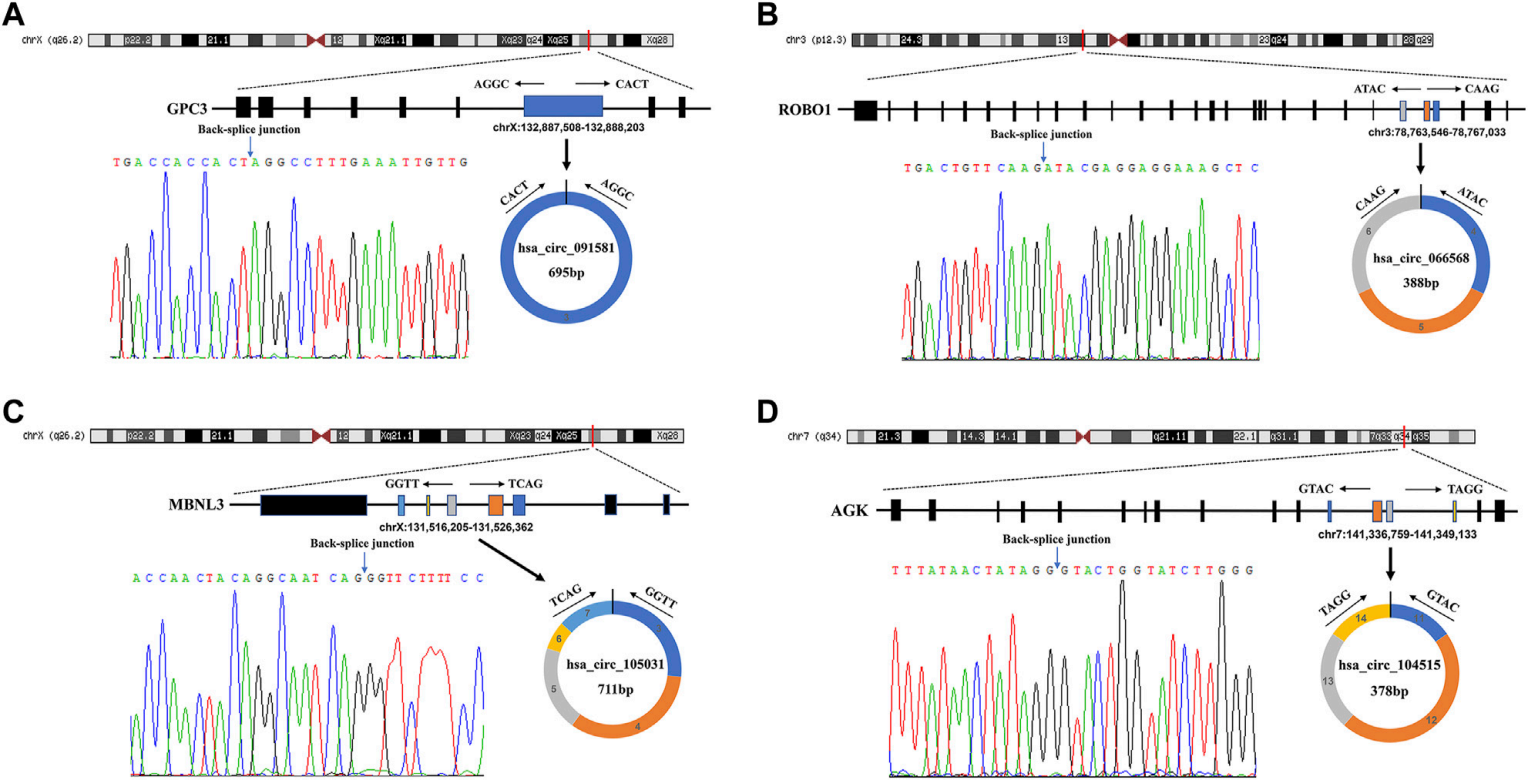
The detailed information of the four circRNAs and their PCR products’ back-splicing junction sites. **(A)** hsa_circRNA_091581. **(B)** hsa_circRNA_066568. **(C)** hsa_circRNA_105031. **(D)** hsa_circRNA_104515. PCR, polymerase chain reaction.

**FIGURE 6:**
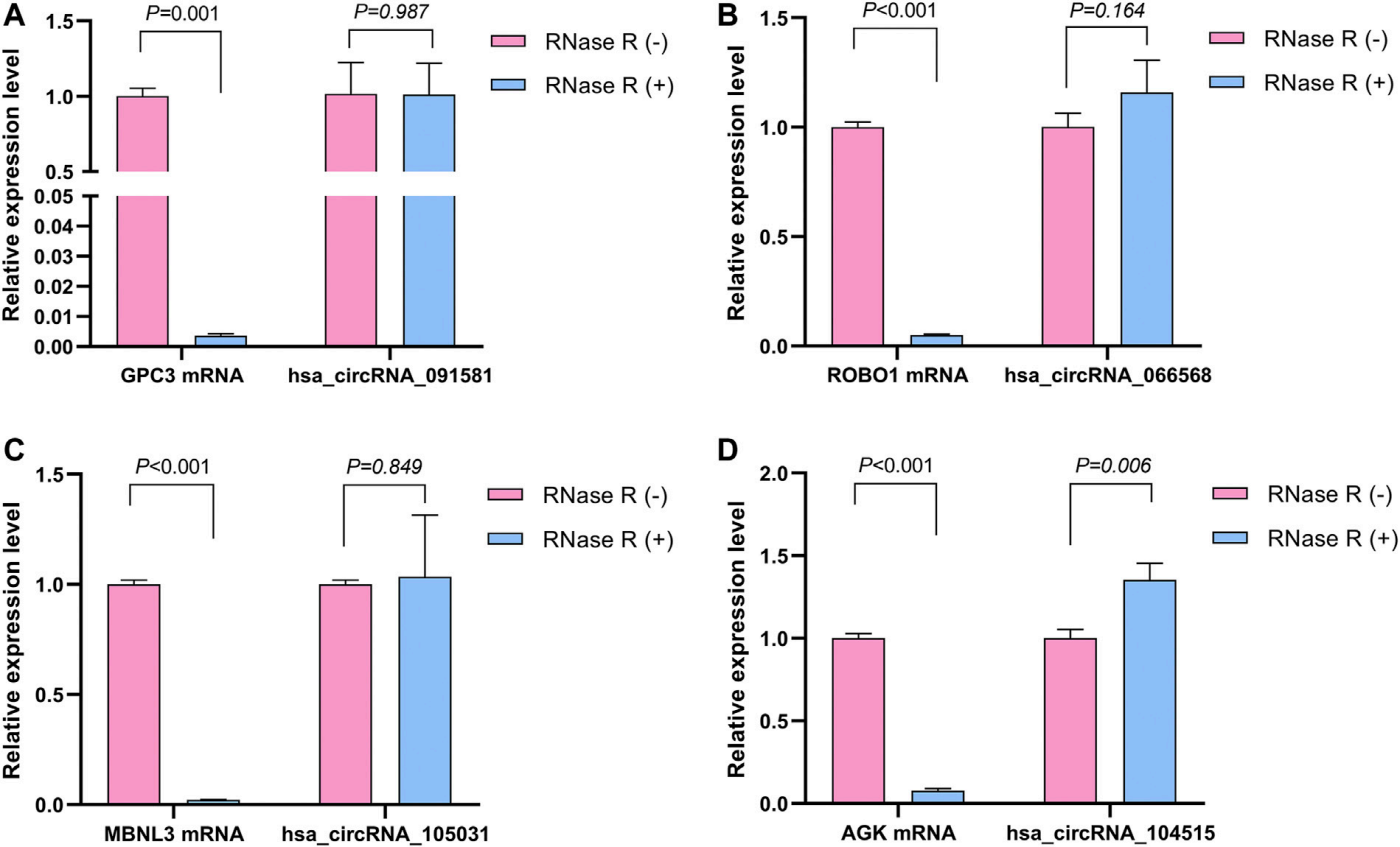
The relative expression levels of the four circRNAs and their corresponding host genes under the RNase R treatment or not. **(A)** hsa_circRNA_091581. **(B)** hsa_circRNA_066568. **(C)** hsa_circRNA_105031. **(D)** hsa_circRNA_104515.

**FIGURE 7:**
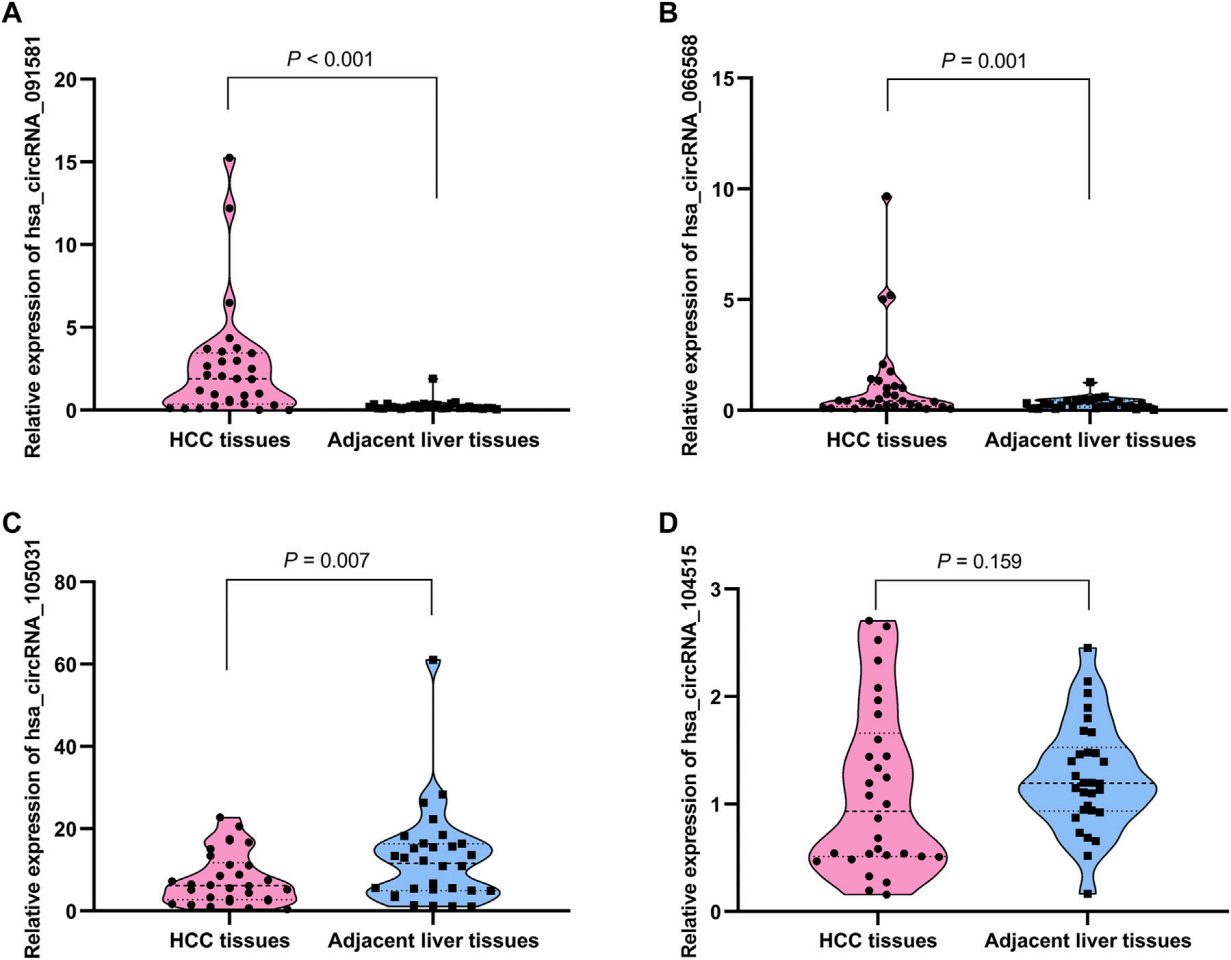
The relative expression levels of the four circRNAs in 30 pairs of HCC tissues and their corresponding adjacent normal liver tissues by qRT-PCR. **(A)** hsa_circRNA_091581. **(B)** hsa_circRNA_066568. **(C)** hsa_circRNA_105031. **(D)** hsa_circRNA_104515. qRT-PCR, quantitative real-time polymerase chain reaction.

The detailed information of the four circRNAs is listed in Table 3 and visualized in Figure 5. Furthermore, the result of TA cloning and sequencing confirmed the back-splicing junction sites of the PCR products of the four circRNAs (Figure 5). To verify the resistance of circRNAs to RNase R digestion, qRT-PCR was performed to evaluate the relative expression of the four circRNAs and their host gene mRNAs under the RNase R treatment or not. As shown in Figure 6, the expression levels of the four mRNAs were significantly reduced in the RNase R (+) group compared to the RNase R (-) group. However, the expression levels of the four circRNAs did not show any obviously downregulated tendency under the RNase R treatment, implying the existence of these four circRNAs. Eventually, the expression levels of the four circRNAs in HCC were examined in 30 pairs of HCC tissues and their corresponding adjacent normal tissues. The results indicated that circRNAs, hsa_circRNA_091581 (*p* < 0.001; Figure 7A), and hsa_circRNA_066568 (*p* = 0.001; Figure 7B) were upregulated and hsa_circRNA_105031 was downregulated in HCC tissues (*p* = 0.007; Figure 7C), which showed statistical significance. However, there was no significant difference in the expression level of hsa_circRNA_104515 between HCC tissues and paracancer tissues (*p* = 0.159; Figure 7D).

**TABLE 3:** The basic information of the four circRNAs identified by bioinformatics analyses.

CircRNA	Alias	Position	Best transcript	Gene symbol	Regulation
hsa_circRNA_091581	hsa_circ_0091581	chrX:132887508-132888203	NM_004484	GPC3	Up
hsa_circRNA_066568	hsa_circ_0066568	chr3:78763546-78767033	NM_002941	ROBO1	Up
hsa_circRNA_105031	hsa_circ_0091570	chrX:131516205-131526362	uc004ewt.3	MBNL3	Down
hsa_circRNA_104515	hsa_circ_0002980	chr7:141336759-141349133	uc003vwi.2	AGK	Down

### Construction of the Preliminary circRNA-miRNA-mRNA Network

**FIGURE 8:**
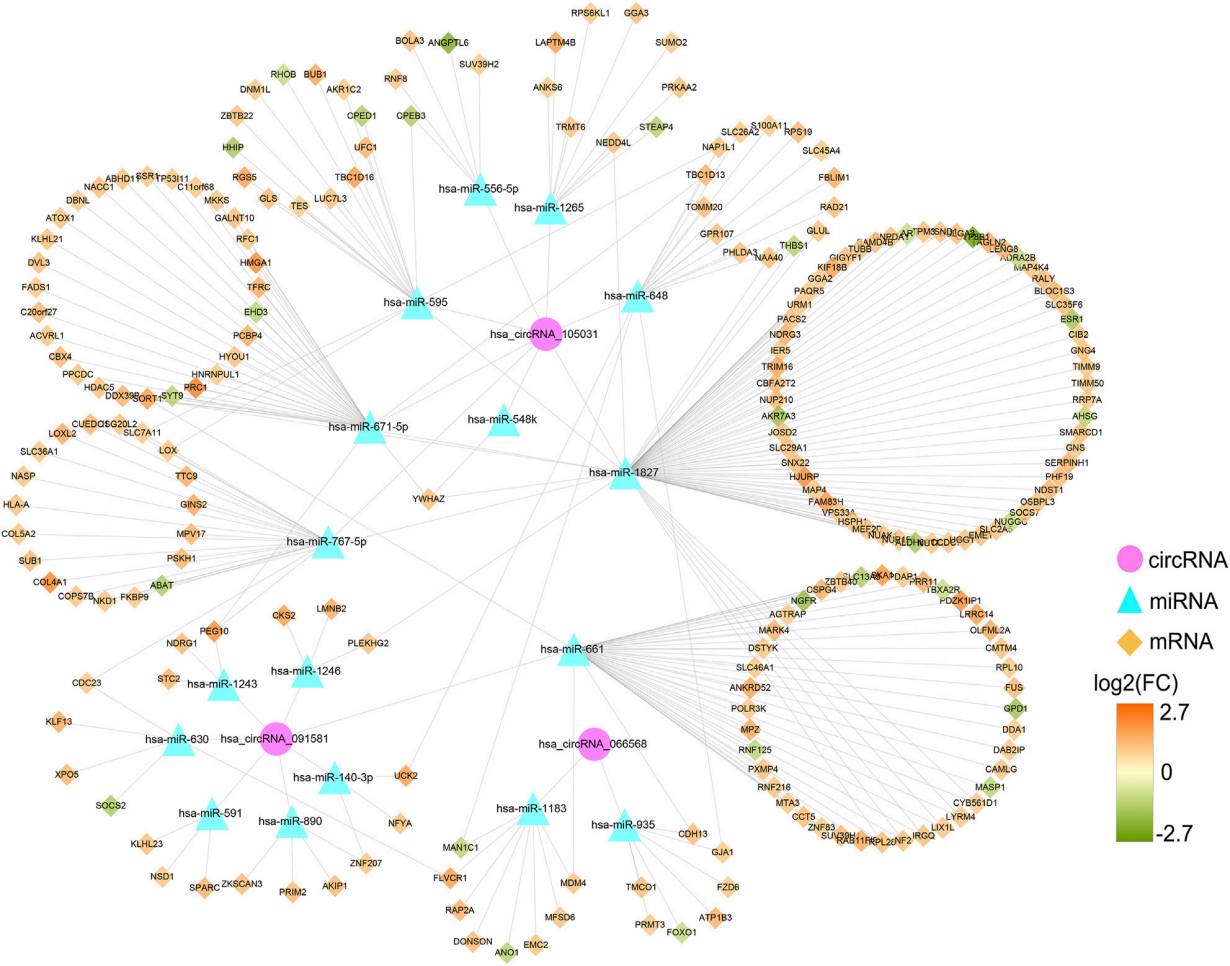
Preliminary circRNA-miRNA-mRNA regulatory network in HCC. The network, consisting of three circRNAs, 17 miRNAs, and 222 mRNAs, was visualized by the Cytoscape software (3.8.3). HCC, hepatocellular carcinoma.

Based on the qRT-PCR results, differentially expressed circRNAs, hsa_circRNA_091581, hsa_circRNA_066568, and hsa_circRNA_105031, were chosen for further analysis. Firstly, we obtained and overlapped the potential target miRNAs of the three circRNAs from two online tools, circBank and CircInteractome. As a result, 17 circRNA-miRNA pairs consisting of three circRNAs and 17 miRNAs were recognized. KEGG analysis of the 17 miRNAs in DIANA-miRPath suggested that these miRNAs were all significantly associated with some cancer-related pathways (Supplementary Figure S1). Next, a total of 1799 predicted mRNAs of these 17 miRNAs were obtained from four miRNA-prediction databases (TargetScan, miRDB, miRTarBase, and miRWalk 3.0). Besides, 2863 DEmRNAs including 2396 upregulated mRNAs and 467 downregulated mRNAs in HCC were found based on the TCGA database (Supplementary Figure S2A). By overlapping the predicted target mRNAs with the DEmRNAs, 222 mRNAs that may exert pivotal effect on HCC were selected for further study (Supplementary Figure S2B). Finally, a preliminary circRNA-miRNA-mRNA regulatory network, which contains three circRNAs, 17 miRNAs, and 222 mRNAs, were constructed based on the above circRNA-miRNA pairs and miRNA-mRNA pairs (Figure 8).

### Identification of Seven Core Genes Based on the Connection Degree of PPI Network

**FIGURE 9:**
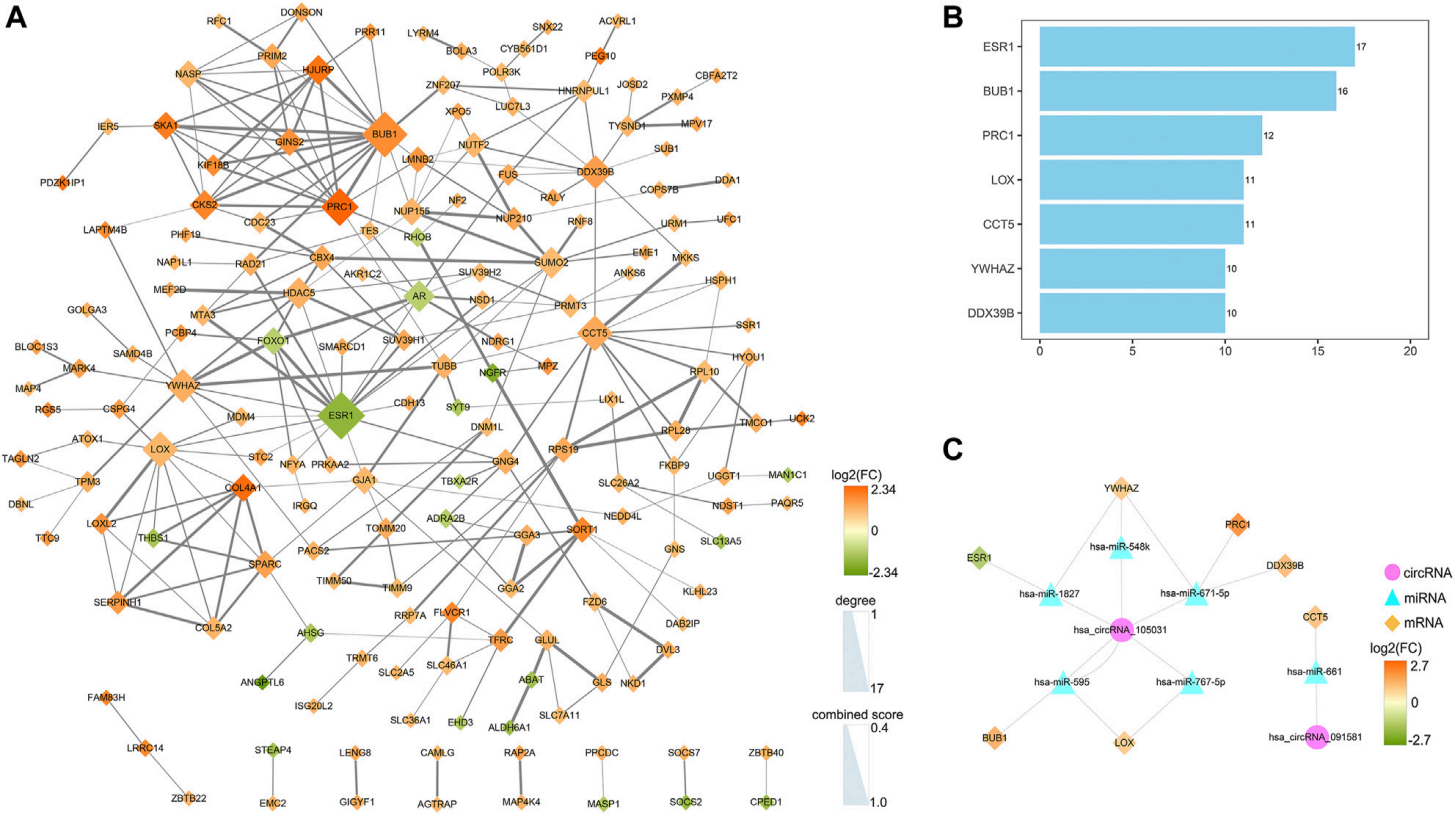
Identification of hub genes from the PPI network and reconstruction of the circRNA-miRNA-mRNA network. **(A)** A PPI network of 222 target genes was analyzed by the STRING (https://www.string-db.org/) online tool and visualized by the Cytoscape software (3.8.3). After removing disconnected nodes, 169 nodes and 269 edges were remained in the PPI network. The node size represents the connection degree with other genes. The size of the edges demonstrates the combined score of two interacting genes. **(B)** Bar plot of the connectivity of the seven hub genes. **(C)** CircRNA-miRNA-hubgene regulatory network in HCC. The network, consisting of two circRNAs, six miRNAs, and seven hub genes, was visualized by the Cytoscape software (3.8.3). PPI, protein-protein interaction.

A PPI network was established to determine the interactions among 222 mRNAs of interest. After removing disconnected nodes, 169 nodes and 269 edges remained in the PPI network and was visualized by the Cytoscape software as shown in Figure 9A. To find out the hub genes involved in the PPI network, the connection degree of each mRNA was calculated by the cytoHubba plugin in Cytoscape. As shown in Figure 9B, seven genes with degree values ≥10 were identified as core genes of the PPI network: *ESR1* (estrogen receptor 1), *BUB1* (BUB1 mitotic checkpoint serine/threonine kinase), *PRC1* (protein regulator of cytokinesis 1), *LOX* (lysyl oxidase), *CCT5* (chaperonin containing TCP1 subunit 5), *YWHAZ* (tyrosine 3-monooxygenase/tryptophan 5-monooxygenase activation protein zeta), and *DDX39B* (DExD-box helicase 39B). Then, a circRNA-miRNA-hubgene regulatory network was reconstructed and visualized in Figure 9C. In summary, ten ceRNA regulatory axes, consisting of two circRNAs (hsa_circRNA_105031 and hsa_circRNA_091581), six miRNAs (has-miR-1827, has-miR-548k, has-miR-671-5p, has-miR-767-5p, has-miR-595, and has-miR-661), and seven hub genes (*ESR1*, *BUB1*, *PRC1*, *LOX*, *CCT5*, *YWHAZ*, and *DDX39B*) were recognized.

### GO Annotation, KEGG, and Reactome Pathway Analyses of the Seven Core Genes

To better understand the potential molecular function of the seven hub genes, enrichment analyses were carried out in the KOBAS-i database and the results are exhibited in Table 4. GO enrichment analysis indicated that the seven hub genes mainly participated in the processes of “protein kinase binding”, “lung development”, “nucleoplasm”, and “protein binding” (corrected *p* < 0.001). KEGG pathway enrichment analysis revealed that the seven hub genes were significantly involved in the “Cell cycle” and “Oocyte meiosis” pathways (corrected *p* < 0.001). As for the Reactome analysis, the seven core genes were enriched in the pathways of “RHO GTPase Effectors” and “Signaling by Rho GTPases” (corrected *p* < 0.001).

**TABLE 4:** Significant GO annotation, KEGG, and Reactome pathways of the seven core genes.

Database	Terms	Input number	Background number	*P*-value	Corrected
					*P*-value
Gene Ontology	protein kinase binding	3	461	5.55E-05	0.006303259
Gene Ontology	lung development	2	83	9.66E-05	0.008238705
Gene Ontology	nucleoplasm	5	3,630	0.000121568	0.008290932
Gene Ontology	protein binding	7	11779	0.000219821	0.009638346
KEGG PATHWAY	Cell cycle	2	124	0.000212439	0.009638346
KEGG PATHWAY	Oocyte meiosis	2	128	0.00022612	0.009638346
Reactome	RHO GTPase Effectors	3	321	1.90E-05	0.006303259
Reactome	Signaling by Rho GTPases	3	449	5.13E-05	0.006303259

GO, gene ontology; KEGG, kyoto encyclopedia of genes and genomes.

### Survival Analysis of the Seven Hub Genes in HCC

**FIGURE 10:**
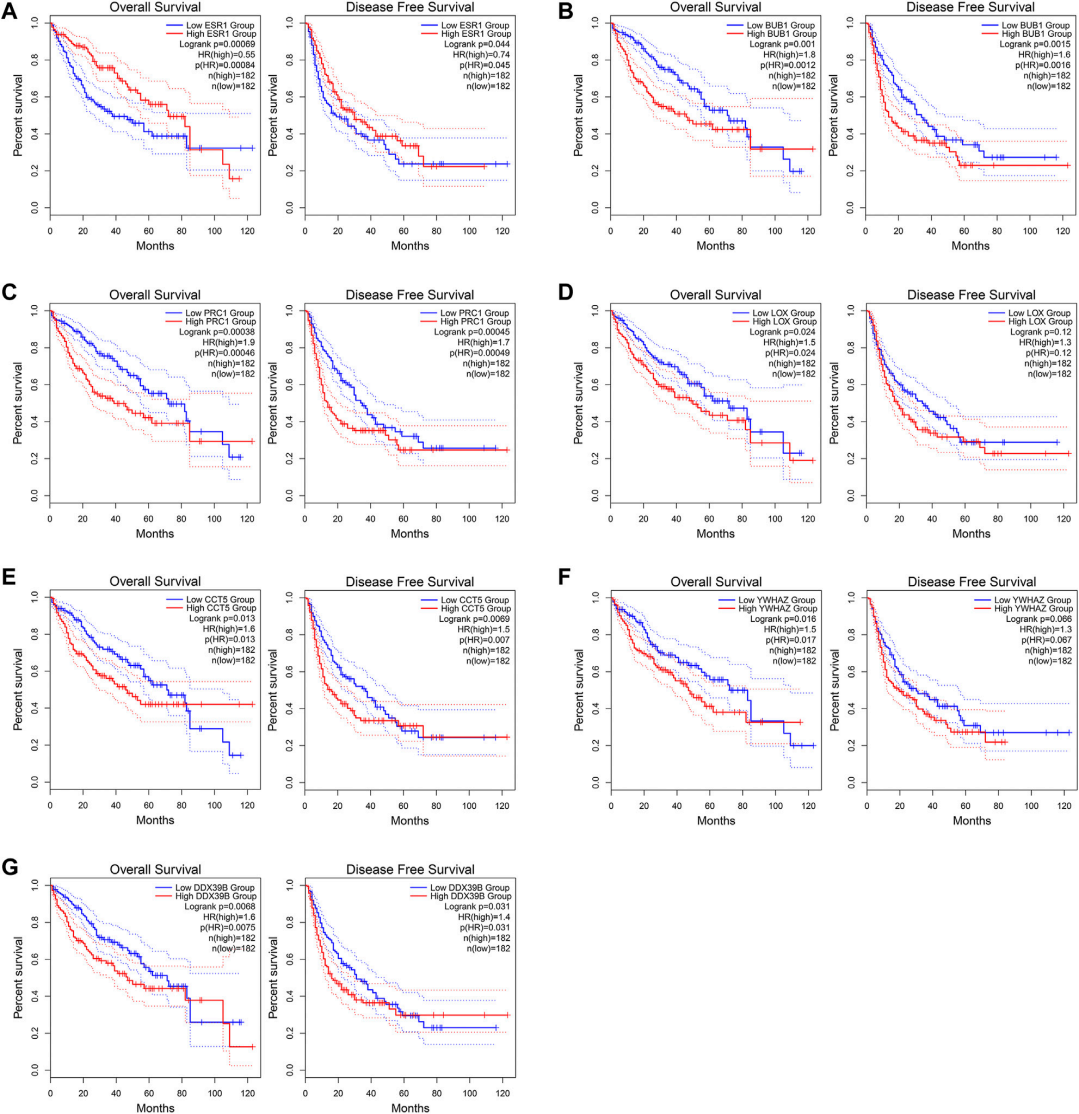
Overall survival (OS) and disease-free survival (DFS) analyses of the seven core genes using the GEPIA2 (http://gepia2.cancer-pku.cn/#index) online tool. **(A)** *ESR1*. **(B)** *BUB1*. **(C)** *PRC1*. **(D)** *LOX* **. (E)** *CCT5*. **(F)** *YWHAZ* **. (G)** *DDX39B*.

The GEPIA2 was utilized to explore the prognostic significance of the seven hub genes in HCC patients and the results are shown in Figure 10. Notably, all the seven core genes were related to the OS of HCC patients based on TCGA database. The high expression of *ESR1* (HR = 0.55) was significantly associated with longer OS of HCC patients (log-rank *p* < 0.05). On the contrary, the high expression of *BUB1* (HR = 1.8), *PRC1* (HR = 1.9), *LOX* (HR = 1.5), *CCT5* (HR = 1.6), *YWHAZ* (HR = 1.5), and *DDX39B* (HR = 1.6) were remarkably correlated with worsened OS of HCC patients (log-rank *p* < 0.05). As for the DFS analysis, the increased expression of *ESR1* (HR = 0.74) was significantly associated with better DFS of HCC patients. The high expression of *BUB1* (HR = 1.6), *PRC1* (HR = 1.7), *CCT5* (HR = 1.5), and *DDX39B* (HR = 1.4) were remarkably correlated with poorer DFS of HCC patients (log-rank *p* < 0.05). However, there was no significant relationship between the expression levels of two core genes, *LOX* (HR = 1.3) and *YWHAZ* (HR = 1.3), and the DFS time of HCC patients (log-rank *p* > 0.05).

### Correlation Between the Hub Genes and Immune Cells in HCC

To further investigate whether the hub genes were related to the infiltrating levels of immune cells in HCC, the TIMER database was applied in our study. As shown in Supplementary Figure S3, the expression levels of *BUB1*, *PRC1*, and *LOX* was significantly associated with the infiltrating levels of six immune cells, including B cells, CD8^+^ T cells, CD4^+^ T cells, neutrophils, macrophages, and myeloid dendritic cells in HCC. The *CCT5* expression level was closely related to the infiltrating levels of all six immune cells except for CD8^+^ T cells in HCC. The *ESR1* expression level was significantly associated with the infiltrating levels of only B cells, CD8^+^ T cells, CD4^+^ T cells, and myeloid dendritic cells in HCC. For *YWHAZ*, the significant positive correlation was observed in B cells, neutrophils, macrophages, and myeloid dendritic cells infiltration in HCC. However, the relationships between *DDX39B* and the infiltrating levels of immune cells in HCC was not explored because *DDX39B* was not available in the TIMER database.

## Discussion

Tumorigenesis is a complex and multi-stage process involving changes in the expression levels of genes and alterations of the microenvironment. CircRNA, a novel type of non-coding RNA, has increasingly become a hot spot of cancer research due to its special covalently closed-loop structure. In this study, four DEcircRNAs in HCC were identified by RRA rank analysis of four GEO datasets (GSE94508, GSE97332, GSE155949, and GSE164803) and WGCNA analysis based on the GSE155949 dataset. Subsequently, the expression levels of these four circRNAs were verified by qRT-PCR and three DEcircRNAs (hsa_circRNA_091581, hsa_circRNA_066568, and hsa_circRNA_105031) were chosen for further analysis. To determine whether the above three circRNAs function as ceRNAs in HCC, a circRNA-miRNA-mRNA regulatory network was constructed based on the predicted targets from online tools and the DEmRNAs from the TCGA database. Seven hub genes (*ESR1*, *BUB1*, *PRC1*, *LOX*, *CCT5*, *YWHAZ*, and *DDX39B*) were recognized in the PPI network and functional enrichment analysis suggested that the core genes were mainly involved in “protein kinase binding”, “Cell cycle”, and “RHO GTPase Effectors” pathways. More importantly, all these seven hub genes were significantly associated with the prognosis of HCC patients and the infiltrating levels of immune cells in HCC. Collectively, the above results indicated that the ceRNA network constructed in this study may play important roles in the tumorigenesis of HCC.

Among the three DEcircRNAs validated by qRT-PCR, hsa_circRNA_091581 was the most studied. Wei et al. found that hsa_circRNA_091581 had an increased expression in HCC tissues, and its high expression was remarkably associated with poor OS and DFS of HCC patients. Mechanism research showed that hsa_circ_091581 can promote HCC cell proliferation by acting as a sponge of miR-526b to block the degradation of *c-MYC* mRNA (Wei X. et al., 2020). Ji et al. also revealed that hsa_circ_091581 promoted HCC progression by targeting the miR-591/*FOSL2* axis (Ji et al., 2021). Besides, as the most upregulated circRNA in the GSE155949 dataset, hsa_circRNA_091581 was experimentally demonstrated to promote the tumorigenesis of HCC by targeting miR-378a-3p to accelerate the cell cycle progression (Han et al., 2021). On the contrary, hsa_circRNA_105031 was found to be downregulated in HCC tissues and suppressed HCC progression by sponging miR-1307 to regulate the expression of *ISM1* (Wang et al., 2019). The above research indicated that the two circRNAs played vital roles in the development and progression of HCC. However, we have not found any relevant studies on hsa_circRNA_066568 yet. Therefore, more studies on the mechanisms of these three circRNAs in HCC are urgently needed, especially for hsa_circRNA_066568.

To further investigate the ceRNA-related mechanism of the three circRNAs in HCC, a circRNA-miRNA-mRNA regulatory network was constructed based on bioinformatics analysis. As a result, three circRNAs, 17 miRNAs, and 222 mRNAs were involved in the ceRNA network. KEGG enrichment analysis of the 17 miRNAs revealed that these miRNAs were involved in multiple pathways related to HCC, such as the Hippo (Liu et al., 2020b), FoxO (Hou et al., 2016), TGF-beta (Mazzocca et al., 2012), PI3K-Akt (Zhou, 2011), and Wnt (Pez et al., 2013) signaling pathways. A PPI network of 222 mRNAs identified seven core genes (*ESR1*, *BUB1*, *PRC1*, *LOX*, *CCT5*, *YWHAZ*, and *DDX39B*), and the enriched GO terms for these hub genes were closely corelated to carcinogenesis processes such as protein kinase binding and protein binding. Cell cycle pathway, the well-known relevant pathway to the aberrant cell proliferation of cancers (Williams and Stoeber, 2012), was the most significant KEGG enrichment pathway of the seven hub genes. For the Reactome pathway analysis, “RHO GTPase Effectors” and “Signaling by Rho GTPases” pathways were also enriched. Previous studies revealed that the RhoGTPases/Rho-effector signaling cascade played important roles in mediating HCC metastasis (Wong et al., 2010). Taken together, the circRNA-miRNA-mRNA regulatory network that we constructed might help to elucidate the regulatory mechanisms of circRNA-related ceRNA in HCC.

In this study, we also constructed the circRNA-miRNA-hubgene network. As a consequence, ten ceRNA regulatory axes (hsa_circRNA_091581/miR-661/*CCT5*, hsa_circRNA_105031/miR-1827/*ESR1*, hsa_circRNA_105031/miR-1827/*YWHAZ*, hsa_circRNA_105031/miR-548k/*YWHAZ*, hsa_circRNA_105031/miR-671-5p/*PRC1*, hsa_circRNA_105031/miR-671-5p/*YWHAZ*, hsa_circRNA_105031/miR-671-5p/*DDX39B*, hsa_circRNA_105031/miR-595/*BUB1*, hsa_circRNA_105031/miR-595/*LOX*, and hsa_circRNA_105031/miR-767-5p/*LOX*), were determined. In addition, the survival analysis based on the TCGA database showed that the expression levels of these seven hub genes were significantly associated with the prognosis of HCC patients, suggesting that the seven hub genes in our network might be promising prognostic indicators for HCC patients. Another interesting finding in our study is that the expression levels of the seven hub genes were closely related to the infiltrating levels of immune cells. Immune cell infiltration, as an important constituent of tumour microenvironment, has been widely considered to be involved in tumour growth and metastasis (Hinshaw and Shevde, 2019). Our study showed that five upregulated hub genes, *BUB1*, *PRC1*, *LOX*, *CCT5*, and *YWHAZ*, had positive relationships with immune cells infiltration levels. In contrast, the downregulated gene, *ESR1*, mainly exhibited negative correlations with immune cells infiltration levels. Given the above evidence, the circRNA-miRNA-hubgene regulatory network may play vital roles in HCC and is worthy of further study.

Existing research have also shown that the seven core genes played important roles in the development and progression of HCC. *ESR1* was identified as a tumour suppressor gene in HCC and its genetic polymorphism was significantly associated with susceptibility to HCC in Chinese hepatitis B virus carriers (Zhai et al., 2006; Hishida et al., 2013). *BUB1*, as a cell cycle related gene, might contribute to the occurrence and development of HCC when there is aberrant increased expression (Yan et al., 2017; Zhang et al., 2020). Chen et al. reported that *PRC1* promoted early HCC recurrence by enhancing the Wnt/β-catenin signaling pathway (Chen et al., 2016). Besides, high *PRC1* expression enhanced HCC cells’ resistance to chemotherapy and was associated with unfavourable survival for HCC patients (Y. Wang et al., 2017). Evidence showed that increased *LOX* could promote angiogenesis, induce epithelial-mesenchymal transition, and is related to early recurrence of HCC (Umezaki et al., 2019; Yang et al., 2019). Furthermore, accumulated evidence has demonstrated that multiple molecules mediated the tumorigenesis and metastasis of HCC by regulating the *YWHAZ*-associated axis (Zhao et al., 2018; Wei et al., 2019; Liu et al., 2020a; Shen et al., 2020). Bioinformatic analysis also revealed the miR-139-5p/*CCT5* axis might exert a momentous effect on HCC progression, but in-depth validated studies have not been carried out (Xu et al., 2021). As for *DDX39B*, its functions in HCC progression have not been studied yet. However, recent studies have revealed that increased *DDX39B* could promote the tumour metastasis of colorectal cancer and enhance chemotherapy resistance in BRCA1-proficient ovarian cancers (Xu Z. et al., 2020; He et al., 2021). In our study, we also found that *DDX39B* was upregulated in HCC tissues and high *DDX39B* expression was correlated to poor OS and DFS in HCC patients based on the TCGA database. These findings suggest that *DDX39B* may function as a tumour promoter in HCC.

Previous studies have also explored the potential circRNA-miRNA-mRNA regulatory networks in HCC. For example, Yang et al. screened out DEcircRNAs based on the GSE97332 dataset and selected the top 10 upregulated and downregulated circRNAs to construct the circRNA-miRNA-mRNA network (Yang et al., 2020). Another study obtained 26 overlapping DEcircRNAs from the GSE97332 and GSE94508 datasets. After being verified to be upregulated in the SMMC-7721 cell line, six DEcircRNAs were selected to construct a circRNA-miRNA-mRNA network (Wang et al., 2020). Besides, Xiong et al. identified six DEcircRNAs by integrating three microarray chips (GSE78520, GSE94508, and GSE97332) using the RRA algorithm. The qRT-PCR results of 16 pairs of HCC tissues and adjacent normal tissues revealed that only has_circRNA_102166 was downregulated in HCC tissues, with a *p*-value less than 0.05 (Xiong et al., 2018). Compared with existing related studies, our research has some advantages. Firstly, we excluded microarray chips with sample sizes less than five pairs and used the RRA method to integrate the four included datasets (N = 67 pairs) to eliminate the effect of small sample size. Secondly, we comprehensively utilized the RRA algorithm, WGCNA analysis, and qRT-PCR validation to screen out critical circRNAs that might play important roles in the progression of HCC. Last but not the least, integrated analyses including PPI network, functional enrichment analysis, survival analysis, and infiltrating immune cells analysis were performed to uncover the potential regulatory mechanisms of HCC progression. However, given that our circRNA-miRNA-mRNA network is constructed based on bioinformatic analysis, further in-depth studies are urgently needed. For example, dual-luciferase reporter and RNA immune co-precipitation (RIP) assays can be performed to verify the interaction between miRNA and circRNA, and between miRNA and mRNA in these predicted regulatory axes. At the same time, more experiments are needed for hsa_circRNA_066568 that has not been reported. Cell counting kit 8, colony formation assay, Transwell migration and invasion assays, cell cycle assay, apoptotic assay, and xenograft tumor assay should be conducted to evaluate the effects of hsa_circRNA_066568 on HCC cells both *in vitro* and *in vivo*. Besides, mechanism researches are also needed to reveal the regulatory axes of hsa_circRNA_066568 in the progression of HCC.

## Conclusion

In summary, our research identified three HCC-related circRNAs (hsa_circRNA_091581, hsa_circRNA_066568, and hsa_circRNA_105031) based on integrated analysis by using the RRA algorithm, WGCNA analysis, and qRT-PCR validation. A circRNA-miRNA-mRNA regulatory network was constructed by computational biology and seven hub genes were identified through the PPI analysis. We also found that the seven core genes might influence the prognosis of HCC patients through regulating the cell cycle and other pathways. Moreover, these hub genes were significantly correlated with the infiltrating levels of immune cells in HCC. Our study provides new insights into the underlying mechanisms of the circRNA-miRNA-mRNA regulatory network in HCC.

## Data Availability

Publicly available datasets were analyzed in this study. This data can be found here: [http://www.ncbi.nlm.nih.gov/geo//GSE94508, GSE97332, GSE155949, and GSE164803] and [https://cancergenome.nih.gov//LIHC project]. The original contributions presented in the study are included in the article/supplementary material, further inquiries can be directed to the corresponding authors.
