# Supplementary figures and images for: Construction and Comprehensive Analysis of a circRNA-miRNA-mRNA Regulatory Network to Reveal the Pathogenesis of Hepatocellular Carcinoma
Source: Front Mol Biosci. 2022 Jan 24;9:801478. doi: 10.3389/fmolb.2022.801478 (PMC8819184; doi:10.3389/fmolb.2022.801478)

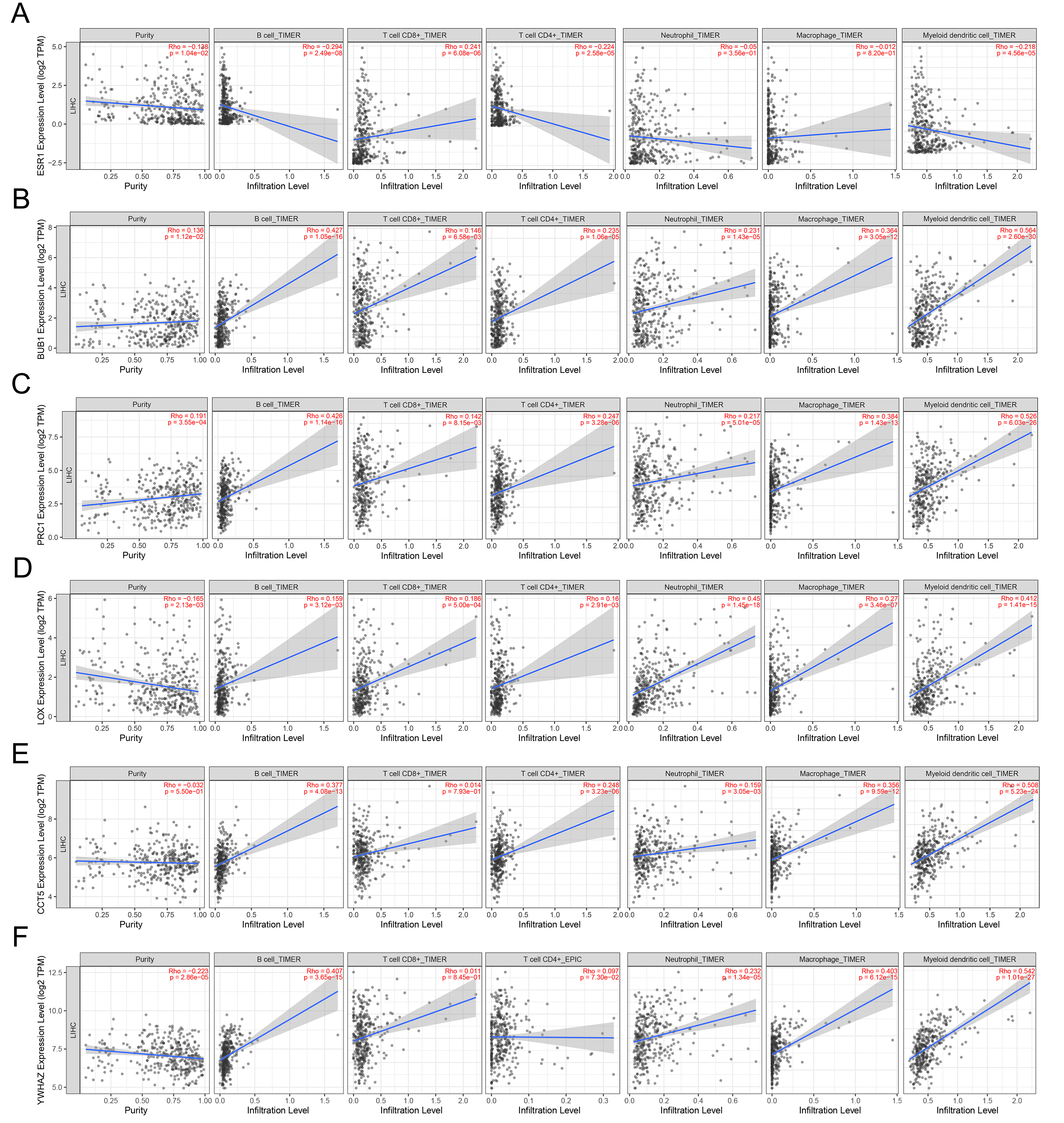

Supplement: Supplementary file 1 [file Image3.TIF]

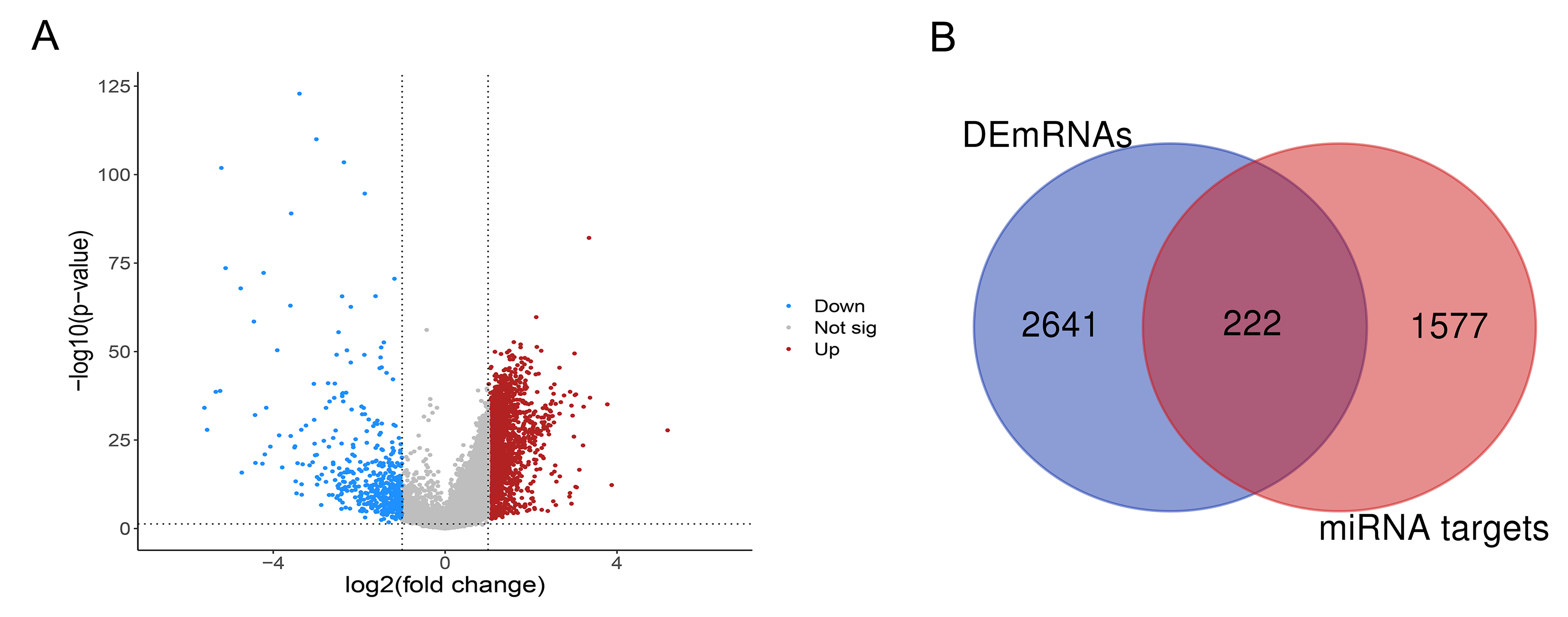

Supplement: Supplementary file 2 [file Image2.TIF]

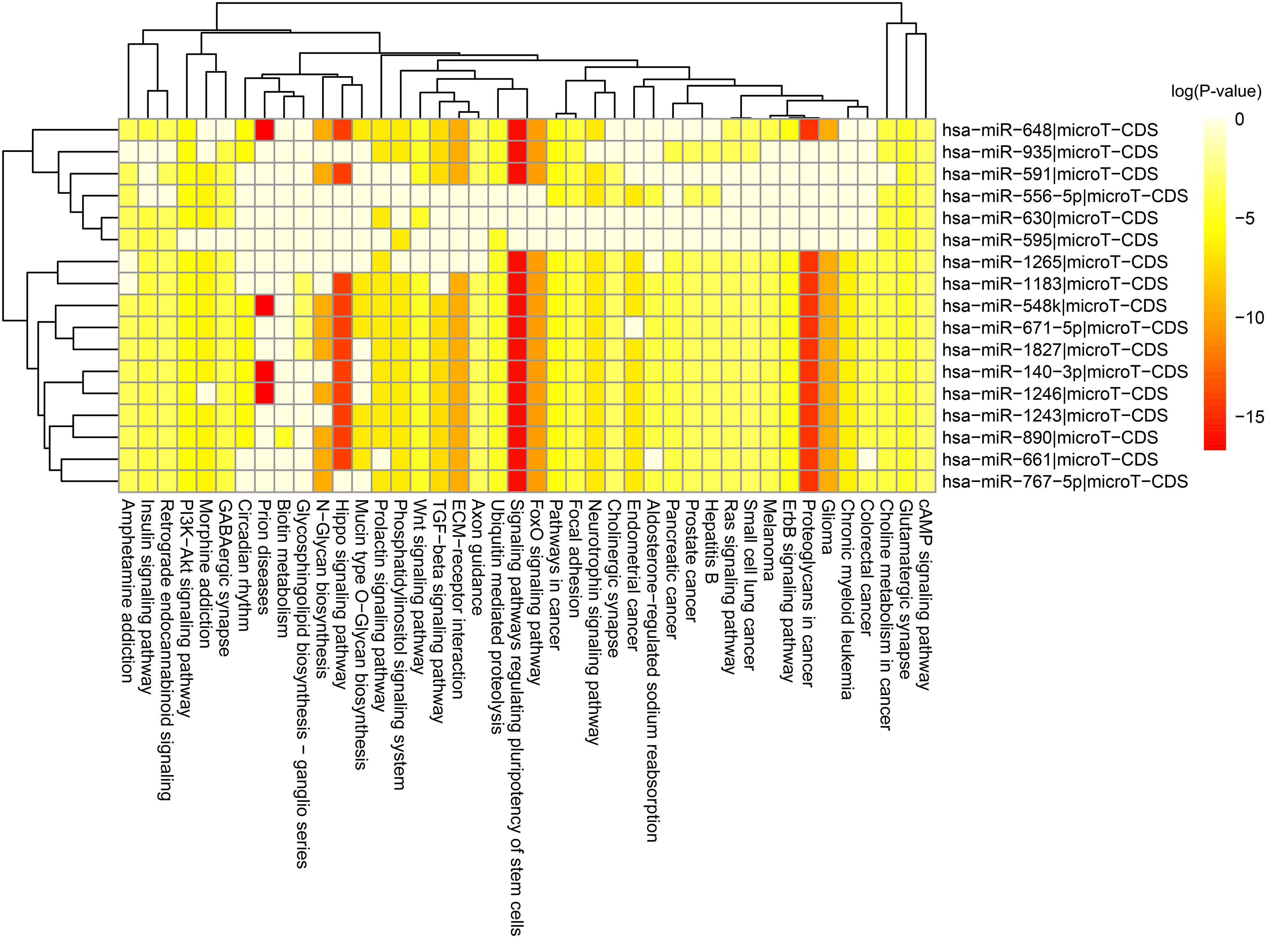

Supplement: Supplementary file 3 [file Image1.TIF]
